# Otitis Media and Obesity—An Unusual Relationship in Children

**DOI:** 10.3390/healthcare9040458

**Published:** 2021-04-14

**Authors:** Cristina Gavrilovici, Elena-Lia Spoială, Anca-Viorica Ivanov, Adriana Mocanu, Violeta Ștreangă, Mirabela-Smaranda Alecsa, Ingrith Miron

**Affiliations:** Pediatrics Department, Grigore T. Popa University of Medicine and Pharmacy, 16 Universitatii Street, 700115 Iasi, Romania; cristina.gavrilovici2012@gmail.com (C.G.); adriana_baltag@yahoo.com (A.M.); streangavioleta@yahoo.com (V.Ș.); subotnicu_mirabela@yahoo.com (M.-S.A.); ingridmiron@hotmail.com (I.M.)

**Keywords:** otitis media, obesity, children

## Abstract

Otitis media (OM) represents a public health matter, being the main cause of preventable hearing loss in pediatric patients. Besides well-established risk factors for developing OM, such as craniofacial abnormalities, prematurity, low birth weight, or tobacco exposure, there is evidence that obesity could be associated with a high incidence of OM. Our aim is to perform a literature review on the state of current published research on the relationship between OM and obesity and to discuss the interconnectivity between these two entities. We conducted an electronic search in PubMed and EMBASE databases. Out of 176 references, 15 articles were included in our study. Our findings suggest that obesity and overweight might be risk factors for developing OM, and vice versa. The main mechanisms for developing OM in obese patients include alteration in cytokine profile, increased gastroesophageal reflux, and/or fat accumulation. Conversely, ear infections exposure might increase the risk of obesity, mostly by taste changes through middle ear cavity inflammation.

## 1. Introduction

Otitis media (OM) represents a public health matter characterized by middle ear inflammation with rapid onset of manifestations, such as pain, fever, anorexia, and irritability [1]. OM represents the second most common cause of primary care admissions in children following respiratory infections with a peak of incidence at 6 to 24 months old [2,3]. About 23% of all infants experience a minimum of one episode of OM, whereas 60% of all children younger than three years of age experience a minimum of one episode of OM, and 24% of them experience a minimum of three episodes [4]. Although the introduction of pneumococcal conjugate vaccines induced a decline in OM incidence, the wide variety of risk factors for ear infections still remains a problem of identification and management [5]. This condition is a leading cause of antibiotic prescription, being an important determinant of preventable hearing loss with negative effects on language, psychosocial, and cognitive development [6].

At the same time, childhood obesity is also a serious concern for the public health nowadays. Globally, in 2016 there were 42 million overweight children under the age of 5, with 31 million living in developing countries [7]. According to Center for Disease Control (US), obesity prevalence in children age 2–19 years old is estimated to be about 13.9% in 2- to 5-year-olds, 18.4% in 6- to 11-year-olds, and 20.6% in 12- to 19-year-olds [8]. There is no consensus on a cut-off point for overweight or obesity in pediatric patients, the body mass index (BMI) being the accepted standard measure for children two years of age and older [9]. In children under 24 months of age, overweight and obesity diagnosis is based on weight-to-length ratio, while for 2–5-year-olds it is based on BMI (using the WHO 2006 and 2007 reference curves) [10,11].

It has been inferred that obesity might contribute to the development of OM through multiple mechanisms such as changes in cytokines levels in host immunity, gastroesophageal reflux, and alteration in function and structure of eustachian tube through adipocytes accumulation [12]. To date, various data have supported the idea that obesity might be a risk factor for OM development. However, recently, it has been hypothesized that there might be a reverse causality as middle ear infections might contribute to a higher predisposition for obesity [13]. Therefore, our goal is to perform a literature review aiming to describe the (potential) relationship between OM and obesity in pediatric patients, in particular whether the obesity is a risk factor for developing otitis media and/or whether OM may predispose to overweight or obesity. We will focus our discussions on three levels: the impact of obesity/overweight on OM development, the impact of OM on the overweight/obesity development, and the mechanism involved in obesity/overweight–OM association.

## 2. Materials and Methods

We performed a systematic literature search on two databases (PubMed and EMBASE), from the time of their initiation to 10 January 2021, to capture original research studies investigating the relationship between obesity and OM in children. We used the following search terms: (“Otitis media”) OR (“ear infection”) OR (“Middle ear infection”) OR AND (“obesity”) OR (“overweight”) AND (“children”) OR (“pediatric”) OR (“paediatric”). We limited our results to humans and performed with restriction to English and French language. All of the significant articles were manually searched to further identify any additional eligible studies.

## 3. Results

### 3.1. Search Results

**Figure 1:**
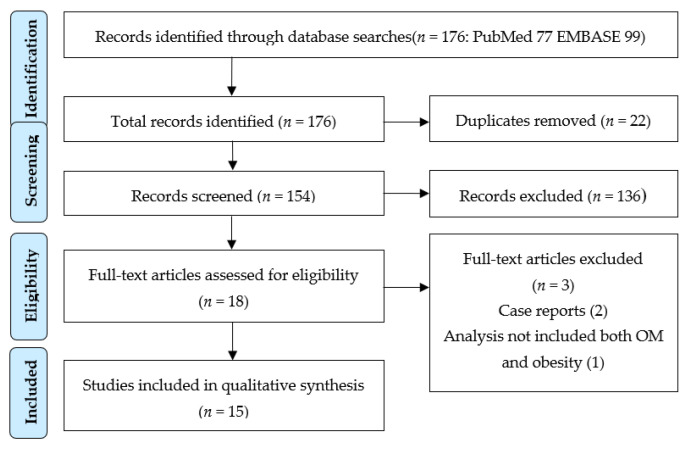
The PRISMA flow diagram for selection of studies for the review (NOTE: “Records” include a mixture of full journal articles of original research, published abstracts, protocols, invited commentaries, and reviews).

The literature search retrieved 176 references (Figure 1). After removing 22 duplicates, 154 titles and abstracts were screened. From the latter, 136 were further excluded due to lack of eligibility criteria (animal studies, manuscripts in a language other than English or French, or non-relevant research results for our purpose: letters to editor, poster presentations, etc.). After reading 18 full text articles, three were excluded as case reports (2) and an analysis that did not include both OM and obesity (1). In total, 15 papers were included in this review.

The final papers had a range of study designs: two cohort studies, six case-control studies, and seven cross-sectional studies. The cohort studies included samples of 42 up to 42.1 million children, aged 1 month–18 years old. Among the seven cross-sectional studies [13,14,15,16,17,18,19], three [14,15,17] were from the United States, and the other four were from the UK [16], Korea [13], Netherlands [18], and Israel [19]. Out of six case-control studies [20,21,22,23,24,25] that we selected, three [23,24,25] were based on the population from Korea, two included children from Turkey [21,22], and one from Saudi Arabia [20].

Of the total of 15 papers included, nine [13,15,17,18,19,20,21,24,25,26] investigated the impact of pediatric obesity on the prevalence and outcome of OM, four papers [14,16,23,27] investigated the hypothesis that ear infections might lead to a higher risk of obesity, and one study [22] suggested that overweight and obesity might be risk factors for developing OM and/or vice versa. A summary of basic characteristics of the included studies is presented in Table 1 (including study, publication year, country, population size, population age, study type, type of ear infection, and methods).

### 3.2. OM—Obesity Relationship: The Impact of Obesity/Overweight on OM Development

Alaraifi et al. [20] evaluated the impact of pediatric obesity on the prevalence and outcome of otitis media with effusion (OME) in 112 children aged 2–18 years. The authors demonstrated that the mean BMI in patients with OME is higher compared with the control group, suggesting that the development of OME may be associated with a higher BMI (19.98 ± 5.20 in OME group versus 17.25 ± 4.21 in control group). The prevalence of obesity was significantly higher in patients with OME compared to the control group (25.0% versus 19.2%). 

In a cross-sectional study, Choi et al. [13] reported that an unbalanced diet with excessive fats intake increased the risk of OME in children. The authors analyzed the association link between OME and dietary fats intake by estimating the intake amount and the proportion of various nutritional components in the diet (total calories, protein, fat, water, and sodium intake). The study included 4359 children aged between 4 to 13 years old. Based on body mass index analysis, a higher fat intake (the percentage of ingested fat calories) was not associated with a higher prevalence of OME in the obese (8.8%) group compared to healthy controls (9.8%), but it was associated with OME in the healthy weight group. 

Moreover, Nelson et al. conducted a cross sectional study [15] on a cohort including 596 infants in order to evaluate whether the children’s weight-for-length (WFL) ≥95th percentile represents a risk factor for developing recurrent OM or for myringotomy tube insertion by the age of two years. In total, 11.4% of eligible enrolled children had a WFL >95th percentile at the age of two years old. Children with a history of tympanostomy tube insertion had a significantly increased weight (WFL ≥ 95th percentile after eliminating the confounding factors such as maternal prenatal smoking, birth weight, or family income) (OR 3.32, 95% CI 1.43–7.72). Recurrent AOM was significantly increased in patients over the 95th percentile (14.8% versus 8.4%), but not in those over the 85th percentile (26.1% versus 19.4%). The results also excluded the reverse causality hypothesis, indicating that infants with higher WFL at the age of two months old would be diagnosed with more OM episodes by two years of age compared to normal weight children. 

Kuhle et al. [26] analyzed the association between obesity and suppurative OM in a prospective cohort study including 3399 children aged between 10–13 years old, among which 788 were overweight and 347 obese. Obese children were more likely to experience OM than normal weight children.

Two case-control studies from Turkey and Korea [21,24] also described the impact of obesity on OM development. Koçyiğit et al. [21] demonstrated on a population sample of 918 children aged between 3 and 15 years old that OME was more frequent among patients with obesity than in healthy controls (17.5 versus. 12.0%). Kim JB et al. [28] conducted a prospective, non-randomized, case-control study [25] in 2007 on 155 children aged two to seven years old who underwent myringotomy tube insertion for OME. The experimental group had a significantly higher BMI compared to the control group (22.0 ± 3.4 versus 16.3 ± 2.4). However, there was no significant difference in myringotomy tube insertion frequency between the obese versus nonobese subgroups. Similarly, four years later, Kim SH et al. [24] conducted a case-control study [27] on a sample of 140 pediatric patients aged 2–7 years old who received unilateral or bilateral myringotomy tube insertion for OME and 190 pediatric patients without OME who underwent various surgical procedures excepting myringotomy tube insertion during the same period; they reported that the frequency of obesity was significantly higher in children with OME. These findings suggest that childhood obesity could be a risk factor for OME development.

In a large cross-sectional analysis including 42.1 million children, Sidell et al. found that 7.0% of school-aged obese children developed acute otitis media (AOM) in contrast to only 4.6% of non-obese patients [17]. These results concur with findings from Shibli et al. [19], which also suggest a link between obesity and a higher rate of OM episodes. On the contrary, Shibli et al. [19] reported that according to weight percentiles, the 85th to 94th percentiles group had fewer cases of acute OM. In obese children (≥95th percentile), the number of observed cases (11) was lower than the number of expected cases (13). The authors of the cross-sectional study [19] conducted in Israel indicated that there were more recurrent admissions than expected in the 95th percentile group for respiratory infections, including OM, which ended up in hospitalization.

Wijga et al. [18] in a cross-sectional study analysis including 3960 8-year-old children reported that there is an association between obesity and respiratory infections, including ear infections. The frequency of ear infection episodes in the previous 12 months was higher in moderate overweight (9.1%) and obese (15.5%) patients compared to normal weight children (9.4%). 

### 3.3. OM—Obesity Relationship: The Impact of OM on the Overweight/Obesity Development

Among the 15 studies included in this review, four papers [14,16,23,27] discuss the possibility that ear infections might lead to obesity and/or overweight. Repeated exposure to ear infections may alter the chorda tympani nerve function which is responsible for taste sensation on the anterior part of the tongue.

A community-based cross-sectional analysis including 485 preschoolers (mean age = 45 ± 7 months) conducted by Peracchio et al. reported that OM exposure frequency was associated with dietary preference and adiposity. The family rated the child’s preference for certain foods (high-fat/added sugar, fruits/juice, vegetables). In the high OM exposure group, fat and sugar foods were the most preferred. Conversely, low OM children ranked fruits or juice as more pleasurable and satisfying than high OM children. A lower affinity for fruits and vegetables and a greater affinity for fat and sugar foods have been observed in the higher BMI percentile group [14]. 

Shin et al. [23] reported a positive association between OME and increased body weight in a study including 42 children between three and seven years old: the mean BMI of the chronic otitis media (COME) group was significantly higher than the control group (20.6 [4.6] versus 17.7 [3.3]). Children who underwent myringotomy tube insertion for OME had a higher BMI than children undergoing other types of surgical interventions. The authors evaluated the changes in taste threshold in patients with COME and their relationship with BMI. It was hypothesized that the inflammatory environment of the middle ear associated with COME may alter the function of the chorda tympani nerve, leading to modifications in taste sensations, food preferences, and consequently BMI. The electrogustometry thresholds were lower in children with OM exposure: the mean thresholds for sweet and salty tastes were significantly higher in the COME group than in the control group. 

Seaberg et al. [27] aimed to evaluate chorda tympani nerve function in a sample of 142 pediatric patients aged between 8 and 18 years old and the link of this function to AOM. The authors hypothesized that the relationship between AOM and obesity may be explained by the alterations in chorda tympani nerve function due to middle ear inflammation. This altered taste function was thought to impact eating habits and result in elevated BMI. According to this study, a higher BMI was not correlated with repeated episodes of AOM. 

Fogel et al. evaluated the link between OM history, adiposity, and the perception of taste in a sample of 196 pediatric patients aged from two to three years old [16]. Their findings showed that higher BMI in five to nine-year-old children was associated with OM history and this could be a consequence of taste impairments. The results showed that boys with a history of repeated OM episodes also had a higher Sucrose Detection Threshold compared to those with a maximum of one episode of OM. In contrast, in two–three-year-old children, OM was not linked to modifications in the perception of taste or BMI, although OM history was associated with higher central adiposity, especially in girls. Kaya et al., in a case-control study conducted in Turkey [22], showed that obesity was more prevalent in children with COME, concluding that obesity and OM might be connected through a bilateral relationship.

## 4. Discussion

In the last decades, there has been increasing evidence for a bidirectional relationship between OM and obesity. Both conditions are significant public health concerns associated with direct and indirect costs. To the best of our knowledge, this review is the first attempt to analyze the link between obesity and OM in children. Although only original studies that directly address the association between ear infections and obesity or overweight were included, the causality relationship between these two conditions was not always clear. Several mechanisms have been described in order to understand the link between OM and obesity. 

Our literature review has gather relevant information to prove that pediatric obesity is associated with a higher prevalence of otitis media with effusion (OME) in children, the mean BMI in patients with OME being higher comparing to healthy, normally developed children. Apparently, only the fat intake is considerably different between the OME and healthy patients, and not total calories. This shows that OME is associated with a fatty diet rather than with higher amounts of food.

Several mechanisms have been described in order to understand the link between OM and obesity. Kim et al. suggested that changes in the cytokines levels, gastroesophageal reflux, accumulation of adipose tissue around Eustachian tube, and also dysfunction of the immune system are among the most relevant explanations for this phenomenon [28]. Studies on pediatric patients with OM confirm that multiple proinflammatory cytokines play a significant role as mediators in ear inflammation. Zielnik-Jurkiewicz et al. [29] reported elevated levels of IL-1β, IL-6, TNF-α and IL-8 in the effusions in pediatric patients with COME. Cottam et al. [30] suggest that due to persistent inflammation in the middle ear and accumulation of adipose cells, obese patients with OM have higher levels of IL-6 and TNF-α than normal weight patients [30]. Moreover, dysfunctions in host immunity associated with obesity, including altered T cell responses, increased secretion of leptin, and interferon γ may explain the increased rates of upper respiratory infections in obese children [31].

Furthermore, a positive association between obesity and gastreoesophageal reflux has been described. It has been found that apparently obesity increases intragastric pressure and transdiaphragmatic gastroesophageal pressure gradient, while the gastric reflux may reach the middle ear through the Eustachian tubes leading to persistent inflammation [32]. An alternative hypothesis suggests that accumulation of adipocytes around the Eustachian tube and nasopharynx may lead to structural alterations and consequently to functional impairment [33,34].

Conversely, repeated exposure to ear infections may be linked to obesity, as some previous studies have reported [14,16,23,27]. 

The existence of severe episodes of OM in childhood was associated with an unbalanced diet consisting of decreased vegetables intake and increased sweets intake, and subsequently to overweight [35]. Moreover, middle ear chronic inflammation may alter the chorda tympani nerve with taste sensation impairment. This can be associated with a higher preference for high-fat products [36]. Alterations in taste sensation may explain why OM patients need a higher ingested quantity of food in order to achieve the same strength of taste as non-OM controls [23]. Recurrent OM patients are more prone to eat sweets and high-fat products rather than vegetables and fruits [14]. Analyzing the studies which approach the relationship between OM and chorda tympani nerve impairment, we have notice that there are apparently discrepant results. While Shin et al. [23] found a link between COME, chorda tympani nerve damage, and consequently BMI changes, Seaberg et al. [27] concluded that AOM episodes did not influence chorda tympani nerve function. This apparently contradictory finding might be explained by age differences (3–7 years old in the study conducted by Shin et al. [23] versus 8–18 years old in the study conducted by Seaberg et al. [27]), or by the duration of inflammation. It seems that chronic inflammation of the middle ear may have a greater influence on chorda tympani nerve function compared to isolated episodes of AOM.

Childhood obesity is a global health issue with both short- and long-term complications. In the United States, the incidence of pediatric obesity increased from 10% in 1999–2000 to 19% in 2015–2016 [37] which parallels the increase of type 2 diabetes in children [38]. According to the Endocrine Society, children with obesity are four times more likely than normal weight children to develop type 2 diabetes [39]. Moreover, Magnus et al., in a prospective study including 99,832 children aged between 4.6–14.2 years old, analyzed the association between pediatric obesity and diabetes mellitus and revealed that obesity diagnosed during the first year of life is positively associated with type 1 diabetes mellitus. This study brings new insight on diabetes mellitus pathogenesis, suggesting that environmental factors might contribute not only to type 2 diabetes mellitus development, but also to type 1 diabetes mellitus [40]. Wernroth et al., in a recent study including a large cohort of 797,318 children, reported a positive association between antibiotic administration for AOM in the first year of life and type 1 diabetes mellitus development before 10 years old (23.8%), especially in children delivered via Cesarean section. The authors suggested that the resilience of the gut microbiota might be decreased in children delivered by cesarean [41].

The research nowadays should be based on moral values and thorough ethical scrutiny [42]. Therefore, some methodological issues should be discussed when interpreting the results of this systematic review. First, the risk of missing out other relevant articles cannot be completely ignored. Secondly, the heterogeneity of the included studies (different age groups and variations in duration of the inflammatory process—acute versus chronic) limited our ability to establish a clear association between OM in general or various types of OM (AOM, OME, COME) and obesity. However, further research is needed, including more prospective studies on all pediatric age groups.

## 5. Conclusions

As we have shown, there are strong evidences for a link between OM and obesity in children. Children with frequent OM episodes may be more prone to obesity, while a higher BMI is a supplementary risk factor for acquiring OM. Although discussed studies shed some light on the mechanisms of obesity and OM, a clear mechanism has not yet been described and further studies are needed in order to understand the pathophysiological processes and to define future strategies for prevention. Moreover, our findings indicate a need to intervene actively by prophylactic measures in order to control both OM and obesity in the pediatric population.

## Figures and Tables

**Table 1:** Summary of basic characteristics of the included studies.

Study, Publication Year, Country	Population Size	Population Age	Study Type	Type of Ear Infection	Methods
Alaraifi, 2020, Saudi Arabia [20]	112	2–18	Case-control study	OME	BMI assessment, data from medical charts and electronic hospital system includinghistory of OME
Fogel, 2019, Birmingham (UK) [16]	196	2–9	Cross-sectional analysis	OM	BMI and waist-to-height ratio assessment, taste sensitivity evaluation (Short Sensory Profile questionnaire), Sucrose Detection Threshold
Koçyiğit, 2017, Turkey [21]	918	3–15	Case-control study	OME	Tympanometry, medical history
Kaya, 2017, Turkey [22]	60	2–10	Case-control study	COME	BMI and waist-to-height ratio assessment, tympanometry
Choi, 2015, Korea [13]	4359	4–13	Cross-sectional study	OME	BMI assessment, medical history, nutritional survey
Peracchio, 2012, United States [14]	485	3–4	Cross-sectional analysis	OM	BMI assessment, medical history, nutritional survey
Shin, 2011, Seoul, South Korea [23]	42	3–7	Prospective, case-control study	COME	BMI assessment, electrogustometry, history of previous AOM episodes
Nelson, 2011, Unitated States [15]	596	2	Cross sectional analysis based on a prospective cohort study	AOM	Waist-to-height ratio assessment, medical history
Kuhle, 2011, Nova Scotia, Canada [26]	3399	10–13	Prospective cohort study	Suppurative OM	BMI assessment, medical history
Seaberg, 2010, Canada [27]	142	5–18	Retrospective cohort study	AOM	BMI assessment, electrogustometry, history of previous AOM episodes
Wijga, 2010, Netherlands [18]	3960	8	Cross-sectional analysis	Ear infection	BMI assessment, questionnaires applied to parents including details about ear infection episodes
Shibli, 2008, Israel [19]	2139	≤13	Cross- sectional analysis	AOM	BMI assessment, questionnaires applied to parents regarding children’s diets and AOM episodes
Kim, 2007, Korea [25]	155	2–7	Prospective, nonrandomized, case-control study	OME	Assessment of BMI, serum triglycerides, and total cholesterol
Sidell, 2013, United States [17]	42.1 million	6–17	Cross-Sectional Analysis	AOM	BMI assessment, medical history
Kim, 2011, Korea [24]	140	2–7	Case- control study	OME	Assessment of BMI, serum triglycerides, and total cholesterol

## Data Availability

No new data were created or analyzed in this study. Data sharing is not applicable to this article.
